# Analysis of Extreme Thermal Variations in the Oral Cavity of a Patient with a Fixed Metallic Orthodontic Appliance Using the Finite Element Method

**DOI:** 10.3390/bioengineering12090901

**Published:** 2025-08-22

**Authors:** Stelian-Mihai-Sever Petrescu, Anne-Marie Rauten, Mihai Popescu, Mihai Raul Popescu, Dragoș Laurențiu Popa, Gabriel Buciu, Eduard-Mihai Ciucă, Tiberius-Cătălin Dudan, Marilena Bătăiosu

**Affiliations:** 1 Department of Orthodontics, Faculty of Dental Medicine, University of Medicine and Pharmacy of Craiova, 200349 Craiova, Romania; mihaipetrescu2702@gmail.com (S.-M.-S.P.); rautenannemarie@yahoo.com (A.-M.R.); 2 Department of Pedodontics, Faculty of Dental Medicine, University of Medicine and Pharmacy of Craiova, 200349 Craiova, Romania; marilena.bataiosu@yahoo.com; 3 Department of Occlusology and Fixed Prosthetics, Faculty of Dental Medicine, University of Medicine and Pharmacy of Craiova, 200349 Craiova, Romania; popescumihairaul@yahoo.com; 4 Department of Automotive, Transportation and Industrial Engineering, Faculty of Mechanics, University of Craiova, 200478 Craiova, Romania; popadragoslaurentiu@yahoo.com; 5 Department of General Nursing, Faculty of Nursing-Târgu Jiu, Titu Maiorescu University, 210102 Târgu Jiu, Romania; 6 Department of Oral and Maxillo-Facial Surgery, Faculty of Dental Medicine, University of Medicine and Pharmacy of Craiova, 200349 Craiova, Romania; ciucaeduard@yahoo.com (E.-M.C.); tiberiusdudan@gmail.com (T.-C.D.)

**Keywords:** cone beam computed tomography, dental tissues, finite element method, fixed metallic orthodontic appliance, thermal variations

## Abstract

Several decades after the development of FEM in computer-based form, which is a milestone in the evaluation of mechanical systems, the method has been adopted to analyze the biomechanical response of human skeletal structures. This innovative technique has generated new questions, but also new results, and, at the same time, competitive environments with explosive development, in the recent period. This research is focused on analyzing, using FEM, the extreme thermal variations produced at the level of two oro-facial systems (one control and one subjected to orthodontic therapy using a fixed metallic orthodontic appliance). The objective of the study was to determine the temperature evolution in different dental structures subjected to extreme temperatures given by variations between very cold and very hot foods. Each system was exposed to a succession of extreme thermal regimes (70…−18…70… °C and −18…70…−18… °C). In order to conduct this research, we used the case of a 14-year-old female patient. Following an orthodontic evaluation, we discovered that the patient had dento-alveolar disharmony with crowding. The straight-wire method of applying a fixed metallic orthodontic appliance was chosen. As complementary examinations, the patient was subjected to a bimaxillary CBCT. Using a series of programs (InVesalius, Geomagic, SolidWorks, and AnsysWorkbench), a three-dimensional model was obtained. This model contained jaws and teeth. Also, brackets, tubes, and orthodontic wires can be incorporated into the model. Following the simulations carried out in this study, it was found that thermal variations from the dental pulp are more severe for the oro-facial system with a fixed metallic orthodontic appliance (regardless of the type of thermal stimulus used). Thus, even today, with all the facilities available in the dental materials industry, metallic orthodontic devices present significant thermal conductivity, generating harmful effects on the dental structures. The reading of the results was performed on the virtual model, more precisely, on the internal dental structures (enamel, dentin, and pulp). A statistical study was not performed because it was considered that, in other patients, the results would be similar.

## 1. Introduction

Malocclusions are characterized by misalignments of the teeth and jaws. Thus, these oral conditions lead to dysfunction of the oro-facial system. The prevalence of malocclusion in children is 79.4% and, for this reason, they are considered a public health problem, affecting the quality of life [1,2].

Cone beam computed tomography (CBCT) represents an innovative alternative to CT investigation, which relies on X-rays to capture internal views of the oro-facial system. Thus, detailed images of these anatomical elements are obtained. CBCT has revolutionized the field of dentistry, being a complementary examination with greater precision than traditional two-dimensional radiographs. All these advantages contribute to establishing the most accurate diagnosis and developing an effective treatment plan [3,4].

In the first decade after the appearance of the finite element method (FEM), this computer-based technique was considered a technique that could solve numerous problems in the field of biomechanics. At the same time, due to the complexity of biological structures, remarkable results were not obtained from the beginning. To this, some phenomena not yet completely elucidated were added, such as friction between bone components, the influence of connective tissues at the periodontal ligament level, the action of muscles, and also the reaction of the human body to the presence of tensions [5,6]. All these disadvantages were successively diminished by the latest results of specialized studies, as well as by improving the method, by using new types of finite elements, and, obviously, by developing hardware systems [7].

In recent times, several decades after the development of FEM in computer-based form, which is a milestone in the evaluation of mechanical systems, the method has been adopted to analyze the biomechanical response of human skeletal structures. This innovative technique has generated new questions, but also new results, and, at the same time, competitive environments with explosive development in recent times [8].

Thus, FEM is a stimulating method, with deep applications in mathematics and engineering, and is also used in various branches of dentistry, including orthodontics.

The different physical and chemical conditions in the oral cavity are believed to greatly affect how natural and artificial structures react, which in turn impacts the overall comfort of the patients. Thus, several studies’ findings indicate that the combined interaction of temperature and pH interferes with the performance of different intraoral devices, including fixed metallic orthodontic appliances [9,10].

The research gap consists of the fact that an experimental technique was not attempted, as it is impossible to place sensors in dental structures such as enamel, dentin, and pulp. This kind of study can only be performed in vitro.

This research is focused on analyzing, using FEM, the extreme thermal variations produced at the level of two oro-facial systems (one control and one subjected to orthodontic therapy using a fixed metallic orthodontic appliance). Each system was exposed to a succession of extreme thermal regimes. The objective of this study is to determine the temperature evolution in different dental structures subjected to extreme temperatures given by variations between very cold and very hot foods. Each system is exposed to a succession of extreme thermal regimes (70…−18…70… °C and −18…70…−18… °C).

## 2. Materials and Methods

The present study was approved by the Ethics Committee of the University of Medicine and Pharmacy of Craiova, Romania (approval reference no. 127/09.04.2024), in accordance with the ethical guidelines of the University of Medicine and Pharmacy of Craiova, Romania, for research with human participants. The legal guardian of the minor patient has given consent for the use of the CBCT package in this study.

For the purpose of this study, we examined a 14-year-old female patient, who presented herself at the Orthodontics Clinic of the Faculty of Dental Medicine of the University of Medicine and Pharmacy of Craiova, Romania.

After an assessment of the orthodontic status, we discovered that our patient had dento-alveolar disharmony with crowding. The straight-wire method of applying a fixed metallic orthodontic appliance was chosen. We opted for this approach because it moves the teeth in sagittal, transverse, and vertical planes, cutting down on treatment time. Modern memory wires, pre-angled brackets, and tubes characterize this technique, allowing sliding tooth movements and guaranteeing safe guidance.

As complementary examinations, the patient was subjected to a bimaxillary CBCT imaging technique performed concurrently for this study. We utilized a CS 8200 3D CT scanner (Carestream Dental, Atlanta, GA, USA). As a result of this investigation, 586 tomographic images were obtained.

The patient’s dento-maxillary apparatus was first visually analyzed using OnDemand3D Dental 1.0.11.1007 (Cybermed Corp., Yusong, Republic of Korea), a medical imaging tool that provided three-dimensional images in addition to the particular equipment. However, obtaining a virtual model that could subsequently be examined using other instruments, like FEM, was not possible with this software.

Consequently, a series of DICOM images was converted into three-dimensional geometries, which are called “point clouds”. These procedures were achieved using InVesalius 3.1.1 (CTI, Campinas, Brazil), an open-source medical research tool, which provides three-dimensional geometries for various tissues based on grayscale.

In order to convert the 3D geometry into precisely closed surfaces, these “point cloud” structures were imported into Geomagic Wrap 2021.2.0 (Morrisville, NC, USA). This engineering software modifies “point cloud” geometric elements. The ultimate objective is to convert these complete surfaces into “virtual solid-type” geometries, which can be imported into a software that uses FEM.

In the next step, the model of precisely closed surfaces was applied to CAD software using SolidWorks 2021 (Dassault Systemes, Velizy-Villacoublay, France). This way, the surfaces are converted into virtual solid models. Orthodontic therapy-specific components (brackets, tubes, and wires) can be incorporated into these models, which are thought to be comparable to the tissues of the patient under analysis. These elements are introduced into the 3D model of the patient under analysis and are specified within a digitally modeled environment having adjustable parameters with the help of CAD techniques. The complete model with perfect geometry is exported to a software that uses FEM.

Ansys Workbench 19.2 (Ansys Inc., Canonsburg, PA, USA) is a program that permits the analysis of a system exposed to a variety of physical phenomena. In the current research, we were interested in the extreme thermal variations produced at the level of two oro-facial systems (one control and one subjected to orthodontic therapy using a fixed metallic orthodontic appliance). The virtual components in this application were given the material characteristics of the biological constituents, as well as the additional components (brackets, tubes, and wires). Ultimately, FEM was used to apply the thermal simulations to the models. Also, data organization and processing were achieved, as well as data production for the virtual models.

In addition to obtaining some figures, graphs, or diagrams, Microsoft Office 2024 (Microsoft Corporation, Redmond, WA, USA) was utilized to organize and analyze the information gathered from the result data provided by the analysis in Ansys Workbench.

The following methods were used in order to produce extreme thermal variations:

Direct engineering techniques, which are specifically featured in SolidWorks 2021, enable the creation of virtual items that resemble real models.

Reverse engineering techniques, which are specifically featured in Geomagic Wrap 2021.2.0, are used to prepare and edit models that were originally made up of so-called “cloud of points”.

Thermodynamics techniques, which are specifically featured in the Ansys Workbench program.

FEM techniques, which are specifically featured in Ansys Workbench 19.2 (AW), represent the core of the mathematical algorithms included in the transient thermal module.

**Figure 1:**
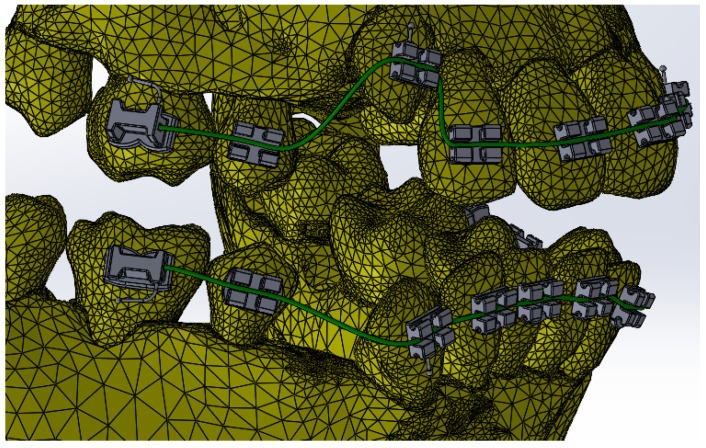
The orthodontic system.

Petrescu S.-M.-S. et al. described the steps of the development of the 3D model of an orthodontic system considered identical to that of the patient. Also, the researchers modeled the metallic orthodontic components of the 3D system using direct engineering techniques and positioned them on the teeth in compliance with the orthodontic guidelines [11]. After the aforementioned steps, resulted the orthodontic system, as shown in Figure 1.

**Figure 2:**
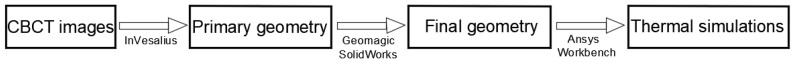
Steps to obtain thermal analysis.

In summary, obtaining a thermal simulation was achieved by using several programs, as shown in Figure 2.

Two categories of simulations were sustained:

The oro-facial system (OFS);
The oro-facial system with metallic braces (OFSMB).

The transient thermal simulation module included in AW was used to load these two models. The control stomatognathic system model is a FEM structure with 1,248,243 nodes and 726,343 elements, while the FEM structure representing the stomatognathic system model with a fixed metallic orthodontic appliance had 1,489,889 nodes and 827,771 elements. This finite element structure is composed of mechanical-type tetrahedra and was obtained automatically, with a target quality of 0.001 m.

The Engineering Data module defined the tissues and materials used in the simulations based on their thermal characteristics. The materials were considered to be isotropic and homogeneous. The utilized tissues and materials are listed in Table 1 [12,13,14].

Next, we defined the temperature sources highlighted at the level of surfaces that are exposed to temperature-diverse foodstuffs.

We chose these values of the thermal stimulus because a hot coffee is normally served at around 70 °C, while the normal temperature of an ice cream is around −18 °C [15,16]. These two foodstuffs represent the extreme temperatures that are frequently consumed by most people around the world.

**Figure 3:**
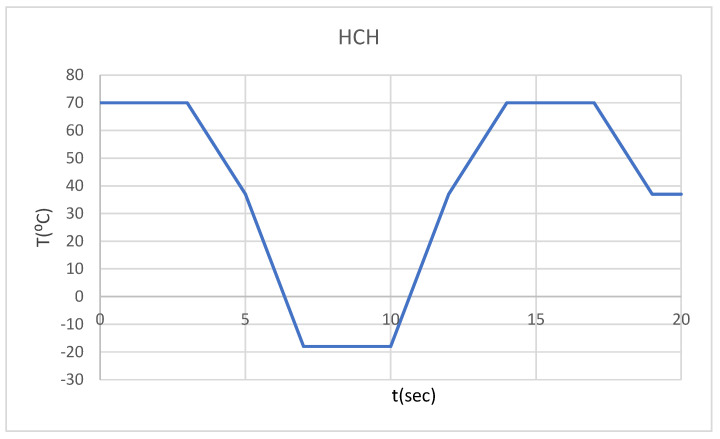
Temperature source regime (HCH).

**Figure 4:**
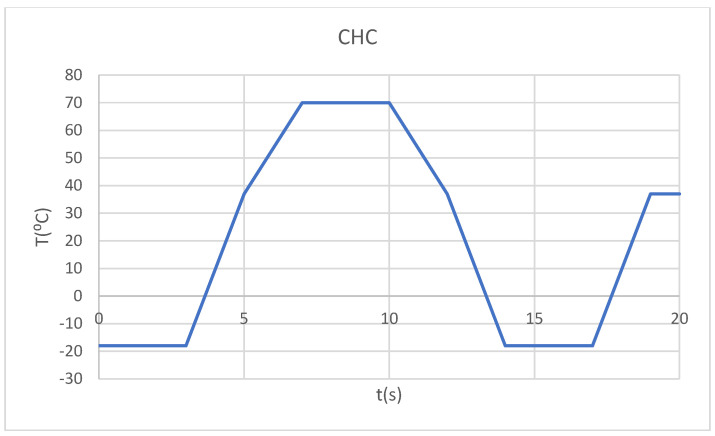
Temperature source regime (CHC).

Thus, we wanted to monitor the behavior of the two systems described previously in the following distinct situations:

- Hot foodstuff (70 °C) for three seconds, followed by cold foodstuff (−18 °C), then hot foodstuff (HCH) (Figure 3);
- Cold foodstuff (−18 °C) for three seconds, followed by hot foodstuff (70 °C), then cold foodstuff (CHC) (Figure 4).

The next step consisted of defining the areas of action of the convection phenomenon. The value that defines the convection phenomenon in the oral cavity is between 2 and 3 W/m^2^ [17].

Petrescu S.-M.-S. et al. selected teeth 1.1 and 4.1 for these virtual thermal simulations. The reason for choosing these dental units was that any external thermal stimulus acting on the dental arches makes initial contact with the upper and lower central incisors [11].

Taking into account the aforementioned details, we used the same teeth to analyze the effects of extreme thermal variations on dental tissues. Thus, to determine the thermal fluctuations at the level of the coronal anatomical structures of 1.1 and 4.1, we used virtual probes of temperature. This operation was possible with the help of the Probe command. The virtual probes of temperature record time-related temperature variations, and the obtained values were centralized in a Microsoft Excel document.

No statistical analyses were performed, as it was considered that, from a thermal point of view, the behavior in other patients is similar.

## 3. Results

### 3.1. Thermal Simulation Results for OFS Subjected Sequentially to HCH Probes of Temperature

**Figure 5:**
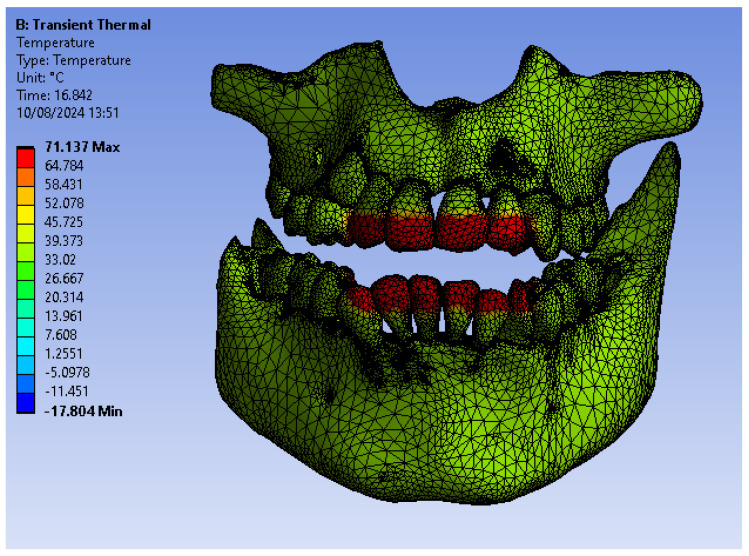
Thermal map.

Figure 5 illustrates the temperature distribution of the system under thermal simulation.

**Figure 6:**
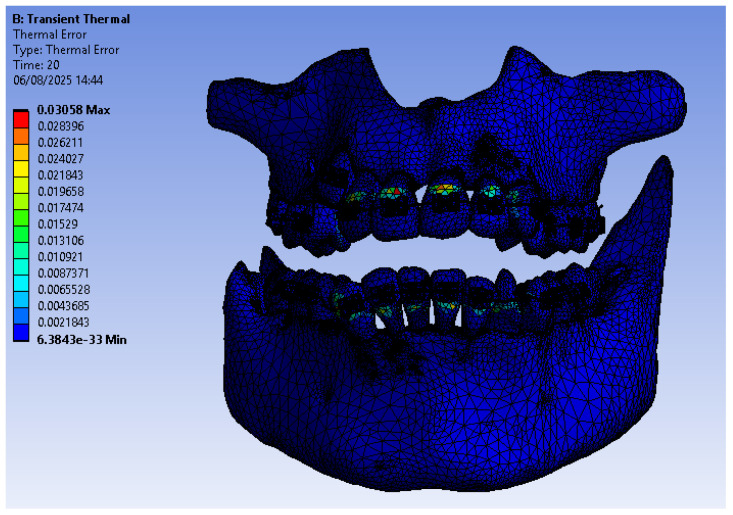
Thermal error map.

In this type of simulation, the thermal error diagram was also presented, as shown in Figure 6. It was found that the maximum error is 0.03058.

**Figure 7:**
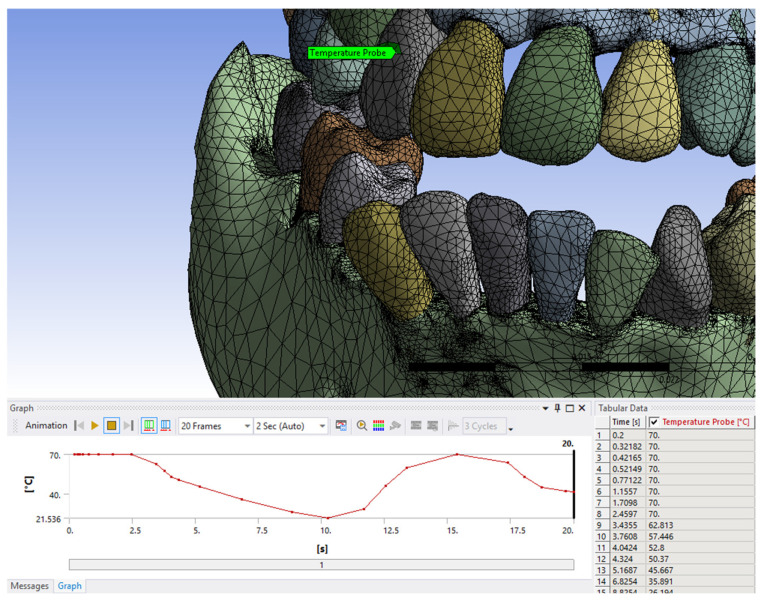
Results in tabular and graphical form.

The temperature value results are shown in data tables similar to those shown in Figure 7. These were imported into Microsoft Excel, and the graphs in the following figures were created.

**Figure 8:**
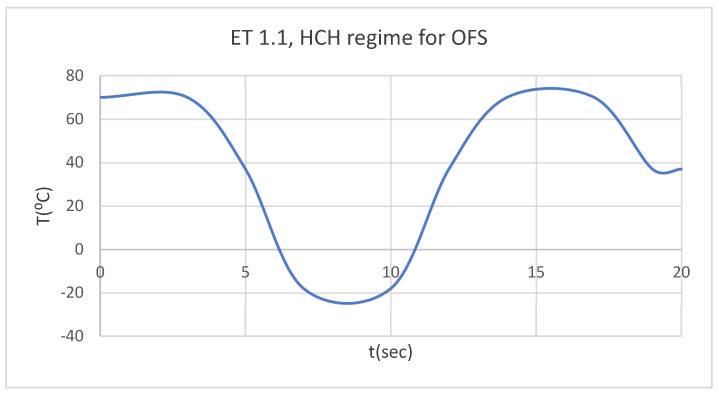
ET for 1.1 subjected to HCH source.

**Figure 9:**
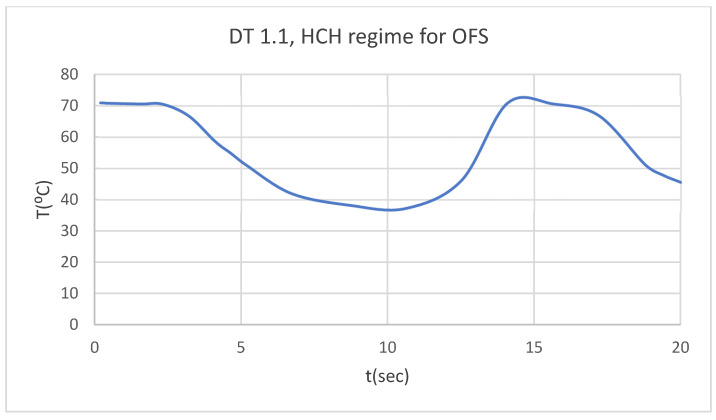
DT for 1.1 subjected to HCH source.

**Figure 10:**
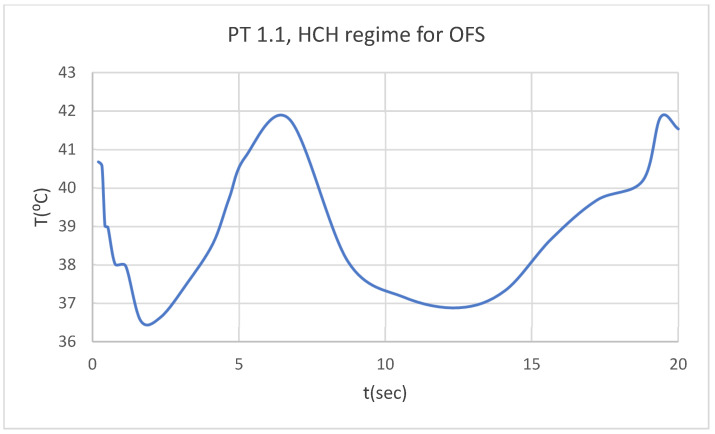
PT for 1.1 subjected to HCH source.

**Figure 11:**
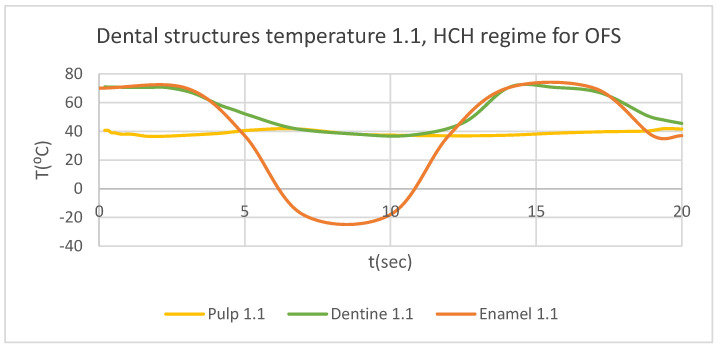
Comparative diagram of the dental structure temperature for 1.1 subjected to HCH source.

**Figure 12:**
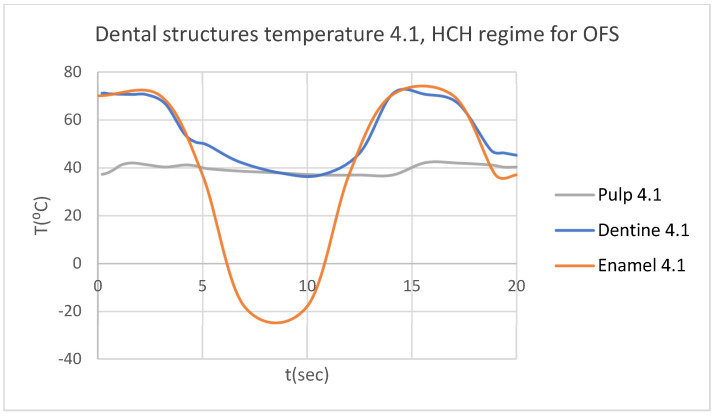
Comparative diagram of the dental structure temperature for 4.1 subjected to HCH source.

**Figure 13:**
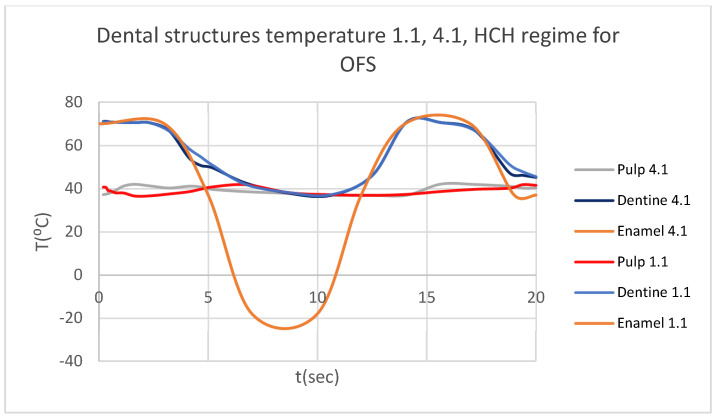
Comparative diagram of the dental structure temperature for 1.1 and 4.1 subjected to HCH source.

Using the Probe-type virtual temperature sensors, we obtained the temperature of the tooth enamel (ET) (Figure 8), dentine (DT) (Figure 9), pulp (PT) (Figure 10), and the comparative diagram of the dental structures for 1.1 (Figure 11). We also obtained ET, DT, PT, and the comparative diagram of the dental structures for 4.1 (Figure 12). Finally, we obtained the comparative diagram of the dental structures for 1.1 and 4.1 (Figure 13).

Similarly to Figure 8, Figure 9 and Figure 10, we also determined each structure temperature (enamel, dentin, and pulp) for 1.1 and 4.1, after subjecting them to HCH and CHC sources.

### 3.2. Thermal Simulation Results for OFS Subjected Sequentially to CHC Probes of Temperature

**Figure 14:**
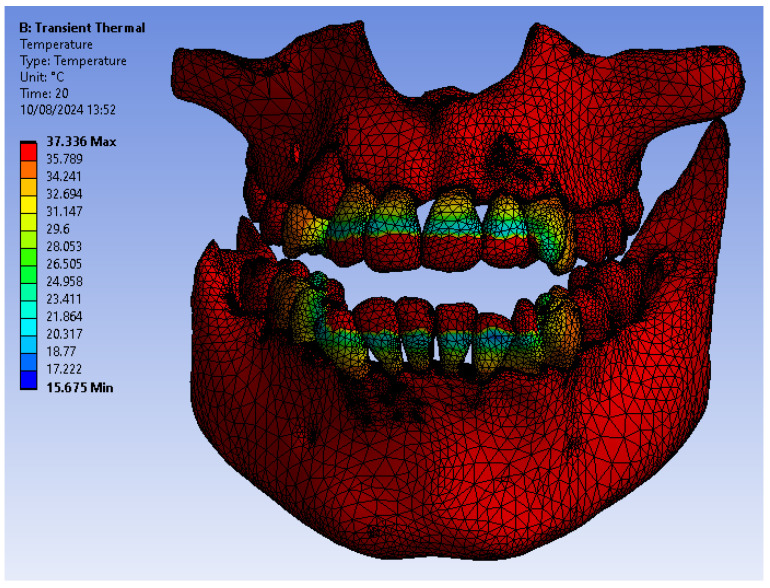
Thermal map.

Figure 14 illustrates the temperature distribution of the system under thermal simulation.

**Figure 15:**
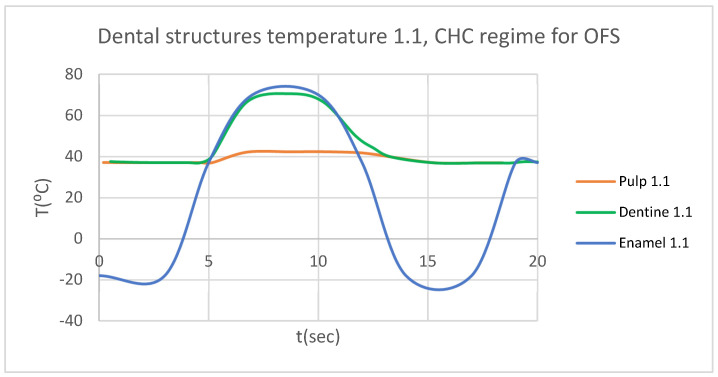
Comparative diagram of the dental structure temperature for 1.1 subjected to CHC source.

**Figure 16:**
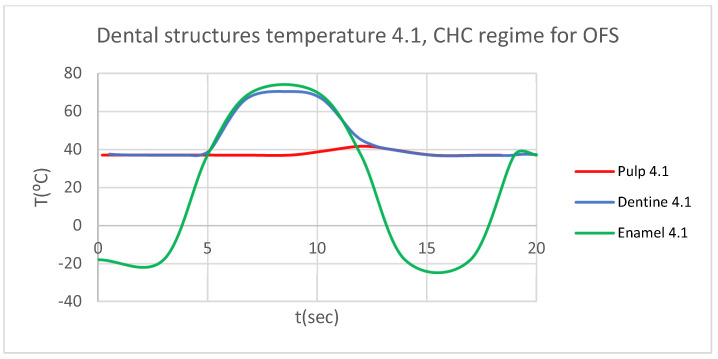
Comparative diagram of the dental structure temperature for 4.1 subjected to CHC source.

**Figure 17:**
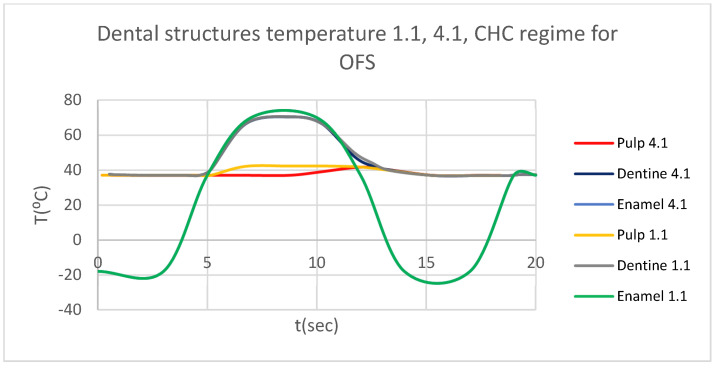
Comparative diagram of the dental structure temperature for 1.1 and 4.1 subjected to CHC source.

Also, we obtained ET, DT, PT, and the comparative diagram of the dental structures for 1.1 (Figure 15). We also obtained ET, DT, PT, and the comparative diagram of the dental structures for 4.1 (Figure 16). We obtained the comparative diagram of the dental structures for 1.1 and 4.1 (Figure 17).

### 3.3. Thermal Simulation Results for OFSMB Subjected Sequentially to HCH Probes of Temperature

**Figure 18:**
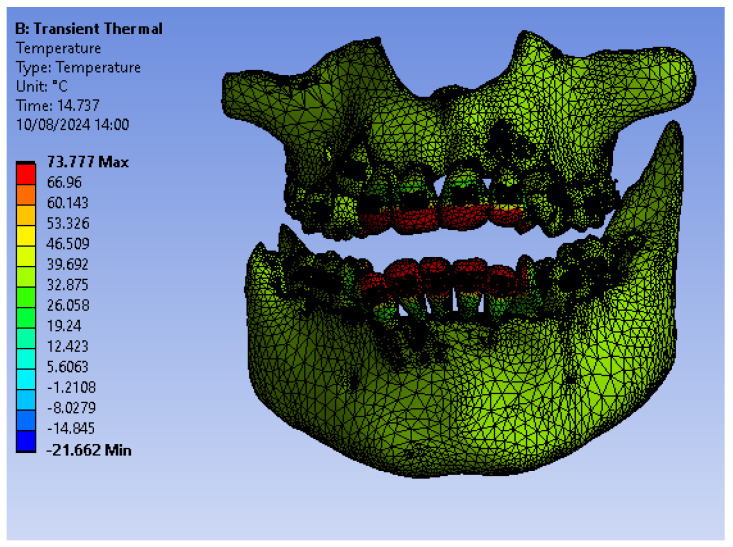
Thermal map.

Figure 18 illustrates the temperature distribution of the system under thermal simulation.

**Figure 19:**
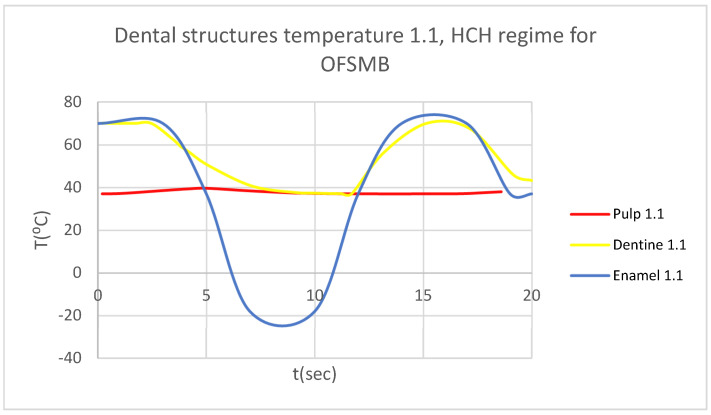
Comparative diagram of the dental structure temperature for 1.1 subjected to HCH source.

**Figure 20:**
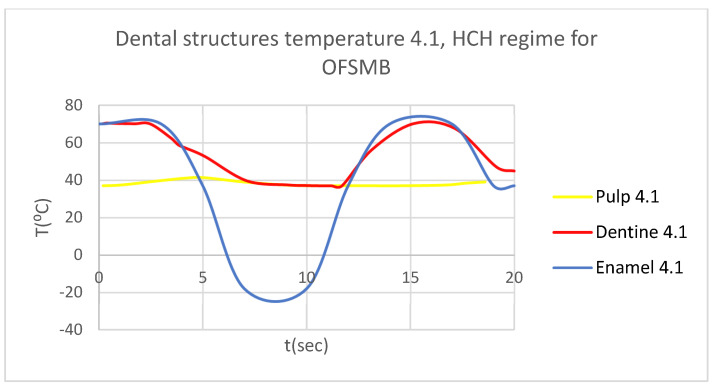
Comparative diagram of the dental structure temperature for 4.1 subjected to HCH source.

**Figure 21:**
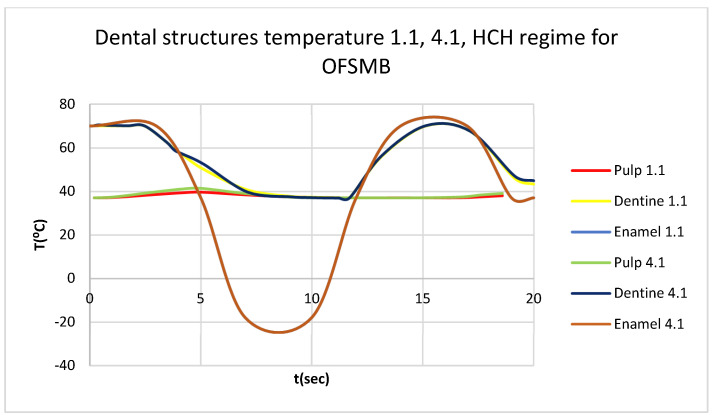
Comparative diagram of the dental structure temperature for 1.1 and 4.1 subjected to HCH source.

In this simulation, we obtained ET, DT, PT, and the comparative diagram of the dental structures for 1.1 (Figure 19). We also obtained ET, DT, PT, and the comparative diagram of the dental structures for 4.1 (Figure 20). Also, we obtained the comparative diagram of the dental structures for 1.1 and 4.1 (Figure 21).

### 3.4. Thermal Simulation Results for OFSMB, Subjected Sequentially to CHC Probes of Temperature

**Figure 22:**
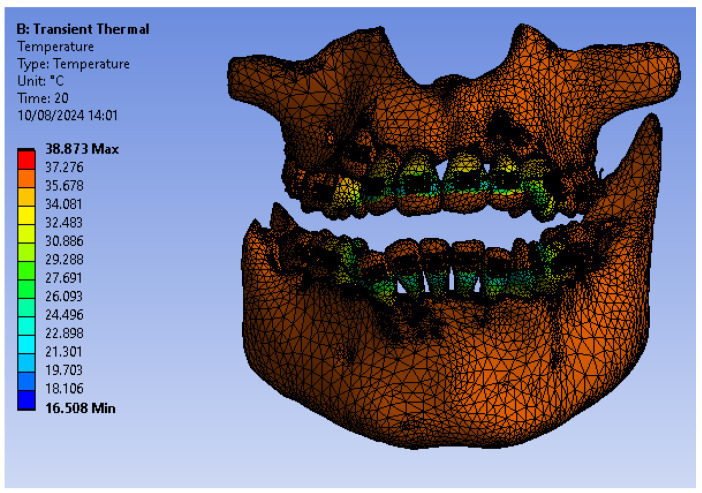
Thermal map.

Figure 22 illustrates the temperature distribution of the system under thermal simulation.

**Figure 23:**
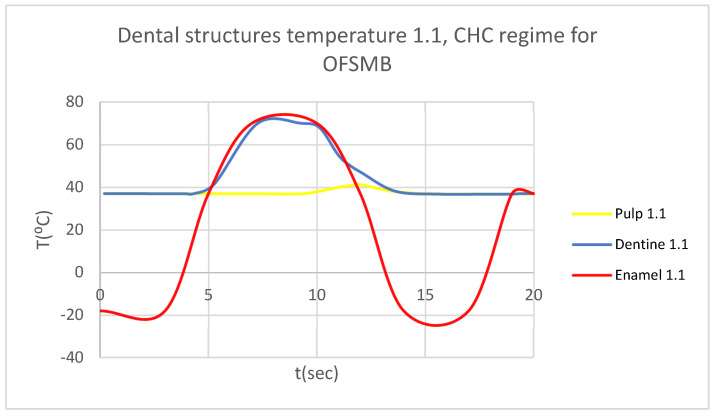
Comparative diagram of the dental structures temperature for 1.1 subjected to CHC source.

**Figure 24:**
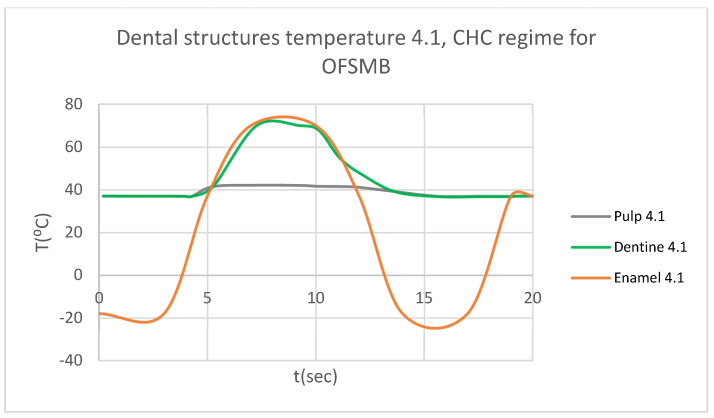
Comparative diagram of the dental structures temperature for 4.1 subjected to CHC source.

**Figure 25:**
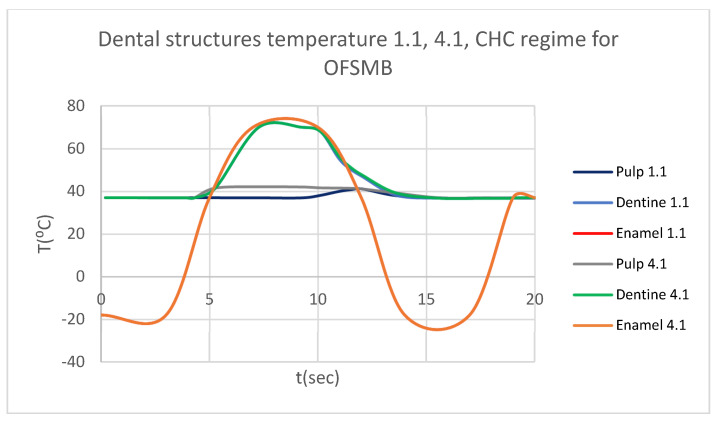
Comparative diagram of the dental structures temperature for 1.1 and 4.1 subjected to CHC source.

In that thermal simulation, we obtained ET, DT, PT, and the comparative diagram of the dental structures for 1.1 (Figure 23. We also obtained ET, DT, PT, and the comparative diagram of the dental structures for 4.1 (Figure 24). In the final, we obtained the comparative diagram of the dental structures for 1.1 and 4.1 (Figure 25).

### 3.5. Comparative Diagrams

According to dental research, an increase or decrease in intrapulpal temperature by approximately 5.5 °C leads to irreversible damage to the pulp tissue [18,19]. Thus, the morpho-functional integrity of the dental pulp is unaffected by thermal changes between 31.5 and 42.5 °C.

**Figure 26:**
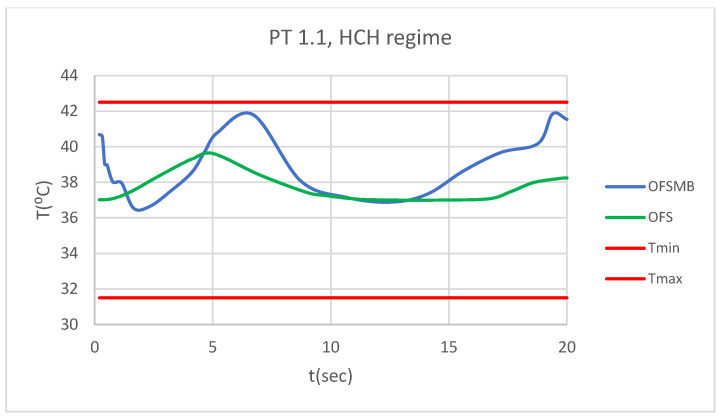
Comparative diagram of how temperature impacts the pulp of 1.1 when subjected to HCH source.

Figure 26 presents a comparative diagram illustrating the effect of temperature on the pulp of 1.1, subjected sequentially to HCH probe.

**Figure 27:**
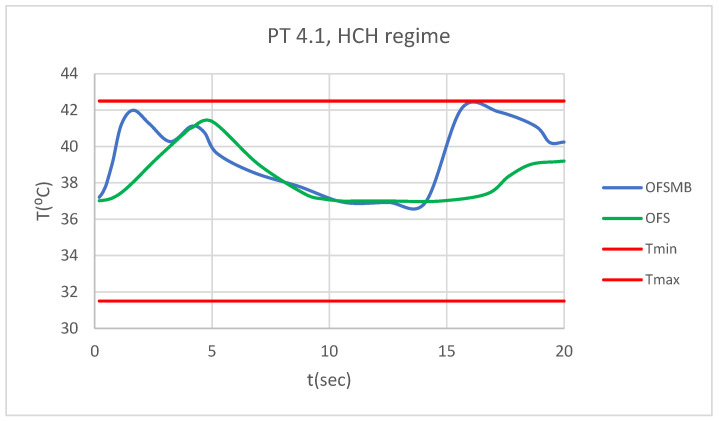
Comparative diagram of how temperature impacts the pulp of 4.1 when subjected to HCH source.

Figure 27 presents a comparative diagram illustrating the effect of temperature on the pulp of 4.1, subjected sequentially to HCH probe.

**Figure 28:**
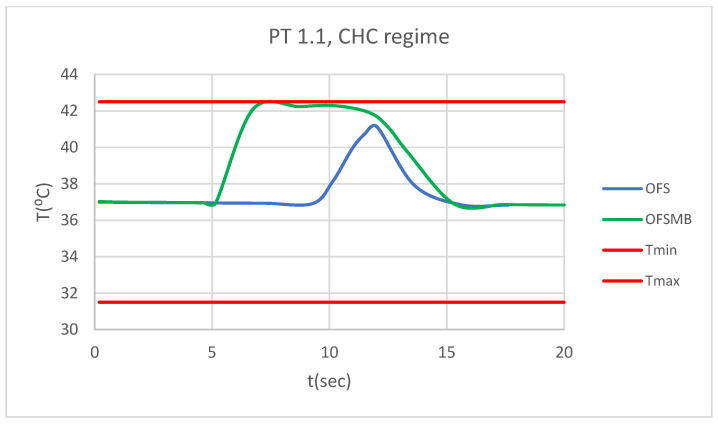
Comparative diagram of how temperature impacts the pulp of 1.1 when subjected to CHC source.

Figure 28 presents a comparative diagram illustrating the effect of temperature on the pulp of 1.1, subjected sequentially to CHC probe.

**Figure 29:**
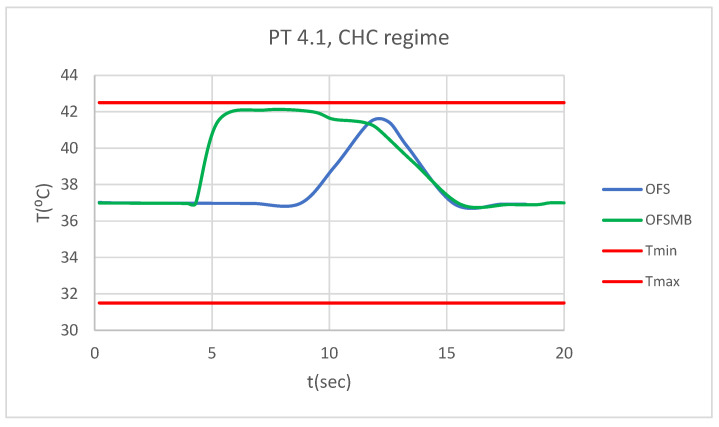
Comparative diagram of how temperature impacts the pulp of 4.1 when subjected to CHC source.

Figure 29 presents a comparative diagram illustrating the effect of temperature on the pulp of 4.1, subjected sequentially to CHC probe.

## 4. Discussion

Thermal studies using FEM in the field of orthodontics are relatively few, as this branch of dentistry mainly focuses on the analysis of the mechanical force distribution on dental tissues, periodontal ligament, and alveolar bone [20,21].

Thermal studies using FEM in orthodontics represent a relatively new field, but with significant promise in terms of deep understanding of the biomechanical and biophysical interactions that occur at the level of orthodontic devices and structures of the dento-maxillary apparatus. The most relevant situations are when the components of the stomatognathic system are affected by different thermal stimuli, such as those generated by the consumption of cold and hot foods and drinks, or by various treatments with external heat sources [22,23].

FEM involves dividing a complex body into small elementary volumes and numerically solving the differential equations that describe the thermal and mechanical behavior inside each element. FEM allows for detailed modeling of heat transfer in structures such as brackets, orthodontic wires, and dental tissues. This approach is particularly valuable because it allows the evaluation of temperature distribution depending on the materials used, the geometry of the orthodontic device components, and the imposed boundary conditions (ambient temperature, saliva contact, etc.) [24].

Thus, FEM can be employed to develop a mathematical representation of the force that occurs as a result of orthodontic wire deformation during specialized treatment, using the straight-wire technique. Forces of different intensities were applied by the researchers, who then measured the resulting displacements at specific locations on the orthodontic wires. Simulations were performed for force intensities (F) from 0.1 to 10 N for both the upper and lower orthodontic wires. The value of the elastic constant (k) was also calculated, according to the relationship k = F/x. According to the authors, the ideal force for effective orthodontic treatment is up to 1 N, as higher forces can cause increased tensions at the root apex and alveolar bone [5].

Another FEM study quantified the extreme stresses, the orientation of applied forces, and the resulting tooth displacements in periodontal tissues with various extents of deterioration. The exerted forces reached a maximum value of 1 N. The main disadvantage of fixed orthodontic treatments is that the generated forces cannot be easily quantified in clinical practice. As with the conclusions of the previously mentioned study, the researchers also advise that force intensity should remain below 1 N for teeth affected by periodontal disease [25].

Regarding the main field of activity of FEM in orthodontics, namely biomechanics, this virtual analysis was also used in the assessment of displacement, strain, and stress at the periodontal ligament level. This anatomical structure plays the most important role during orthodontic therapy, mediating the processes of bone resorption and apposition responsible for tooth displacements. Taking into account the data from the specialized literature, the researchers used forces between 0.5 and 1 N during the simulations. As a result, the evolution of the diagrams depending on the force applied to the metallic orthodontic components was almost linear. In addition to their inherent rigidity, the orthodontic systems demonstrated elasticity, resulting from the properties of the orthodontic wires and the periodontal ligaments [6].

A similar experimental study using FEM described the physical effects resulting from the action of distinct thermal agents on an OFS and on an OFSMB. Following the action of thermal stimuli on the virtual models, with the help of the AW program, based on FEM analysis, the authors concluded that temperatures are significantly higher at the pulp of OFSMB [11].

There are several fields in dentistry, except orthodontics, which use finite element analysis, such as endodontics. Similarly to orthodontists, endodontists use FEM to assess dispersions of stress and variations in temperature. An important aspect in FEM studies from the field of endodontics, different from those in the field of orthodontics, is the evaluation of endodontically treated teeth. Thus, a study used the PubMed database to compare the results of 30 publications and concluded that FEM is a useful method in endodontics for analyzing dispersions of stress and variations in temperature [26].

Another study uses a finite element model in conjunction with related studies to examine dynamic heat exchange in the oral cavity under various situations. This work investigated temperature distribution across several oral zones as well as transient thermal dynamics by modeling and measuring intraoral temperature during cold drink consumption, temperature recovery, and continuous cold air inhalation. The findings show that while premolars and molars sustain higher and more stable temperatures after being exposed to cold, incisors and canines show greater thermal sensitivity and a slower rate of temperature recovery. Furthermore, buccal zones are typically warmer and more stable than the lingual ones, highlighting how crucial measurement location is to accuracy [27].

Regarding the weaknesses of this study, we can refer to the lack of a thin film of orthodontic adhesive used for the bonding of bracket and tube-type elements. Modern composites, including those used in fixed orthodontic treatments, have physical properties similar to those of dental hard tissues, which theoretically does not influence the transmission of thermal stimuli to the dental pulp. Also, as previously mentioned, a thin film of adhesive is located between the enamel surface and the base of the brackets and tubes, the thermal isolation effect being reduced or even absent. Another disadvantage present in the case of thermal simulations performed at the level of the oral cavity using FEM is represented by the absence of saliva. Saliva can produce an attenuation of the thermal effect of various stimuli, helping to regulate the intraoral temperature. However, our study was conducted at the dental pulp level of 1.1 and 4.1, these being the first teeth to come into contact with foods and drinks, with minimal salivary protection.

The discrepancies between the pulp reaction of 1.1 and 4.1 exist because the two aforementioned teeth are morphologically different. Therefore, 1.1 is more voluminous, with better-represented enamel and dentin layers. These hard dental tissues protect the dental pulp from all harmful external stimuli, including thermal ones. Also, enamel and dentin have reduced thermal conductivity, opposite to metallic materials.

As future research directions, we propose to analyze with the help of FEM the effect of different thermal agents (hot drinks) on clear aligners, in the event that the patient does not remove them from the oral cavity. According to studies in the specialized literature and manufacturers’ indications, aligners should be removed from the oral cavity before consuming any type of drink or food. We will also consider future studies in which more patients will be analyzed. Research that includes salivary flow can be performed in AW, using Fluid Flow modules, to determine the Convection Coefficient. Then, the results obtained can be used in Thermal Transient.

## 5. Conclusions

The analysis of the resulting data led to the conclusion that, for 1.1, the highest pulp temperatures occurred in the CHC regime, both with and without metallic braces. In contrast, for 4.1, the highest pulp temperatures were observed in the HCH regime, regardless of the presence of braces.

Subsequent to the simulations executed in this study, it can be observed that the thermal variations from the dental pulp are more severe in the case of OFSMB (regardless of the type of thermal stimulus used). Thus, even today, with all the facilities available in the dental materials industry, metallic orthodontic devices present significant thermal conductivity, generating harmful effects on dental structures. Through studies of this kind carried out with the help of FEM, it is possible to achieve an improvement in the manufacturing technologies of orthodontic devices made of different metal alloys, as well as the adjuvant consumable materials used in orthodontic therapy.

Also, the presence of a fixed metallic orthodontic appliance determines the retention of bacterial plaque, which is often hard to remove with usual oral hygiene techniques. As it is known, bacterial plaque contributes to the appearance of acids, which initiate enamel demineralization. The combination of a fixed metallic orthodontic appliance and the presence of enamel demineralization or caries represents a threat to the vitality of the pulp, which is more easily affected by thermal and chemical variations in the oral cavity.

FEM studies on thermal variations in orthodontics represent a niche but increasingly important area of research. This can be concluded from the fact that the extremely varied temperature in the oral cavity influences not only the tissues that compose the anatomical structures, but also the materials used in orthodontic treatments.

## Figures and Tables

**Table 1:** Thermal properties of the tissues and components used in simulations.

Component	Density [Kg/m^3^]	Isotropic Thermal Conductivity [W·m/°C]	Specific Heat [J·Kg/°C]
Enamel	2958	0.93	710
Dentine	2140	0.36	1302
Pulp	1000	0.0418	4200
Maxillary bones	2310	1	2650
Ni + Cr alloy (bracket and tube-type elements)	8500	13	460
Ni + Ti alloy (orthodontic wires)	6450	60	457

## Data Availability

The authors declare that the data from this research are available from the corresponding authors upon reasonable request.
